# Validation of the myocardial-ischaemic-injury-index machine learning algorithm to guide the diagnosis of myocardial infarction in a heterogenous population: a prespecified exploratory analysis

**DOI:** 10.1016/S2589-7500(22)00025-5

**Published:** 2022-04-20

**Authors:** Dimitrios Doudesis, Kuan Ken Lee, Jason Yang, Ryan Wereski, Anoop S V Shah, Athanasios Tsanas, Atul Anand, John W Pickering, Martin P Than, Nicholas L Mills, High-STEACS Investigators, Nicholas L Mills, Fiona E Strachan, Christopher Tuck, Anoop SV Shah, Atul Anand, Andrew R Chapman, Amy V Ferry, Kuan Ken Lee, Dimitrios Doudesis, Anda Bularga, Ryan Wereski, Caelan Taggart, Matthew TH Lowry, Filip Mendusic, Dorien M Kimenai, Dennis Sandeman, Philip D Adamson, Catherine L Stables, Catalina A Vallejos, Athanasios Tsanas, Lucy Marshall, Stacey D Stewart, Takeshi Fujisawa, Mischa Hautvast, Jean McPherson, Lynn McKinlay, Ian Ford, David E Newby, Keith AA Fox, Colin Berry, Simon Walker, Christopher J Weir, Alasdair Gray, Paul O Collinson, Fred S Apple, Alan Reid, Anne Cruikshank, Iain Findlay, Shannon Amoils, David A McAllister, Donogh Maguire, Jennifer Stevens, John Norrie, Jack PM Andrews, Alastair Moss, Mohamed S Anwar, John Hung, Jonathan Malo, Colin Fischbacher, Bernard L Croal, Stephen J Leslie, Catriona Keerie, Richard A Parker, Allan Walker, Ronnie Harkess, Tony Wackett, Roma Armstrong, Laura Stirling, Claire MacDonald, Imran Sadat, Frank Finlay, Heather Charles, Pamela Linksted, Stephen Young, Bill Alexander, Chris Duncan

**Affiliations:** a British Heart Foundation Centre for Cardiovascular Science, University of Edinburgh, Edinburgh, UK; b Usher Institute, University of Edinburgh, Edinburgh, UK; c Department of Non-communicable Disease Epidemiology, London School of Hygiene and Tropical Medicine, London, UK; d Department of Emergency Medicine, Christchurch Hospital, Christchurch, New Zealand; e Christchurch Heart Institute, Department of Medicine, University of Otago, Christchurch, New Zealand

## Abstract

**Background:**

Diagnostic pathways for myocardial infarction rely on fixed troponin thresholds, which do not recognise that troponin varies by age, sex, and time within individuals. To overcome this limitation, we recently introduced a machine learning algorithm that predicts the likelihood of myocardial infarction. Our aim was to evaluate whether this algorithm performs well in routine clinical practice and predicts subsequent events.

**Methods:**

The myocardial-ischaemic-injury-index (MI^3^) algorithm was validated in a prespecified exploratory analysis using data from a multi-centre randomised trial done in Scotland, UK that included consecutive patients with suspected acute coronary syndrome undergoing serial high-sensitivity cardiac troponin I measurement. Patients with ST-segment elevation myocardial infarction were excluded. MI^3^ incorporates age, sex, and two troponin measurements to compute a value (0–100) reflecting an individual's likelihood of myocardial infarction during the index visit and estimates diagnostic performance metrics (including area under the receiver-operating-characteristic curve, and the sensitivity, specificity, negative predictive value, and positive predictive value) at the computed score. Model performance for an index diagnosis of myocardial infarction (type 1 or type 4b), and for subsequent myocardial infarction or cardiovascular death at 1 year was determined using the previously defined low-probability threshold (1·6) and high-probability MI^3^ threshold (49·7). The trial is registered with ClinicalTrials.gov, NCT01852123.

**Findings:**

In total, 20 761 patients (64 years [SD 16], 9597 [46%] women) enrolled between June 10, 2013, and March 3, 2016, were included from the High-STEACS trial cohort, of whom 3272 (15·8%) had myocardial infarction. MI^3^ had an area under the receiver-operating-characteristic curve of 0·949 (95% CI 0·946–0·952) identifying 12 983 (62·5%) patients as low-probability for myocardial infarction at the pre-specified threshold (MI^3^ score <1·6; sensitivity 99·3% [95% CI 99·0–99·6], negative predictive value 99·8% [99·8–99·9]), and 2961 (14·3%) as high-probability at the pre-specified threshold (MI^3^ score ≥49·7; specificity 95·0% [94·6–95·3], positive predictive value 70·4% [68·7–72·0]). At 1 year, subsequent myocardial infarction or cardiovascular death occurred more often in high-probability patients than low-probability patients (520 [17·6%] of 2961 *vs* 197 [1·5%] of 12 983], p<0·0001).

**Interpretation:**

In consecutive patients undergoing serial cardiac troponin measurement for suspected acute coronary syndrome, the MI^3^ algorithm accurately estimated the likelihood of myocardial infarction and predicted subsequent adverse cardiovascular events. By providing individual probabilities the MI^3^ algorithm could improve the diagnosis and assessment of risk in patients with suspected acute coronary syndrome.

**Funding:**

Medical Research Council, British Heart Foundation, National Institute for Health Research, and NHSX.

## Introduction

Myocardial infarction is a condition characterised by myocardial necrosis secondary to acute myocardial ischaemia, and is the most common cause of death worldwide.^1^ In recognition of this, clinical guidelines emphasise the importance of early diagnosis and treatment to reduce mortality, and clinicians have a low threshold for referring patients for further investigation.^2^ However, although patients with suspected myocardial infarction account for one in 20 attendances in the emergency department,^3^ the diagnosis is ultimately ruled out in 80% to 90% of patients.^4^, ^5^

Accelerated diagnostic pathways aim to promote earlier discharge in patients considered low-risk and improve the targeting of treatment to patients at high-risk.^6^, ^7^, ^8^, ^9^ However, these pathways have some limitations. First, they use fixed cardiac troponin thresholds for all patients, which do not account for age or comorbidities that are known to influence troponin concentrations.^9^, ^10^ Second, they are based on fixed time-points for serial testing, which can be challenging in a busy emergency department, and such pathways might not be generalisable to all health-care systems. Third, up to one third of patients are neither ruled-out, nor ruled-in, using these pathways and questions often remain for these individuals. For example, how probable was it that the patients' symptoms were due to a heart attack, and would they benefit from further testing?

**Research in context**

**Evidence before this study**

Patients with suspected myocardial infarction account for approximately one in 20 attendances in the emergency department. The myocardial-ischaemic-injury-index (MI^3^) is a machine learning algorithm that predicts the likelihood of myocardial infarction in patients with suspected acute coronary syndrome. We systematically searched PubMed for studies published up to Jan 18, 2022, using the following keywords: “machine learning”, “myocardial infarction”, and “troponin” with no language restrictions. Three machine learning algorithms were identified from this search but none that had used high-sensitivity cardiac troponin to predict the likelihood of myocardial infarction.

**Added value of this study**

This is the largest study evaluating the diagnostic performance of a machine learning algorithm for the diagnosis of myocardial infarction and the first to be performed in a consecutive patient population that reflects clinical practice. The MI^3^ algorithm had excellent overall discrimination. We observed no heterogeneity in our subgroup analysis for the low-probability threshold, and the performance was heterogenous across subgroups for the high-probability threshold. Moreover, we report for the first time that patients identified as high-probability by the algorithm of myocardial infarction on the index visit also had a ten-times higher rate of subsequent myocardial infarction or cardiovascular death at 1 year than patients who were classified as low-probability.

**Implications of all the available evidence**

Our findings have potentially important implications for the use and interpretation of this algorithm in clinical practice. MI^3^ could improve the diagnostic pathways for myocardial infarction by accurately identifying patients at high risk of myocardial infarction to be targeted for prompt individualised treatment, and by allowing early discharge in patients at low risk.

The myocardial-ischaemic-injury-index (MI^3^) is an algorithm developed using the machine learning technique, gradient boosting, to compute an individualised probability of myocardial infarction on a scale of 0–100 for patients with suspected acute coronary syndrome.^11^ The MI^3^ score is computed using age, sex, cardiac troponin concentration, and the rate of change in troponin concentration when re-measured at a second flexible time point. Although the algorithm performed well when validated in data pooled from seven diagnostic cohort studies of patients with suspected acute coronary syndrome,^11^ it has not been evaluated in a more heterogeneous patient population, in which a greater burden of comorbid conditions might affect performance. Furthermore, it is not clear whether the algorithm provides information about cardiovascular risk beyond the initial diagnosis of myocardial infarction.

In consecutive patients with suspected acute coronary syndrome, we evaluate whether MI^3^ can predict the index diagnosis of myocardial infarction and risk of subsequent myocardial infarction or cardiovascular death at 1 year.

## Methods

### Participants

High-sensitivity troponin in the evaluation of patients with suspected acute coronary syndrome (High-STEACS) is a stepped-wedge cluster randomised controlled trial that evaluated the implementation of a high-sensitivity cardiac troponin I (hs-cTnI) assay in consecutive patients with suspected acute coronary syndrome, across ten secondary and tertiary care hospitals in Scotland, UK.^12^ All adult patients (age >18 years) with suspected acute coronary syndrome attending the emergency department were identified by the attending clinician at the time troponin was requested, using an electronic form integrated into the clinical care pathway. For this prespecified exploratory analysis of the trial, patients were eligible for inclusion if they presented with suspected acute coronary syndrome and had at least two serial cardiac troponin measurements. Patients were included from both the assay validation and implementation phases of the trial. Patients were excluded if there was insufficient clinical information to adjudicate the diagnosis, or if they presented with ST-segment elevation myocardial infarction because patients with this presentation was not included in the original development of the algorithm.

The High-STEACS trial was approved by the Scotland A Research Ethics Committee, the Public Benefit and Privacy Panel for Health and Social Care, and by the National Health Service (NHS) Health Board for each hospital. As randomisation was at the hospital level, consent was not sought from individual patients. All data were collected prospectively from the electronic patient record, deidentified and linked to regional and national registries in a data repository within a secure NHS Safe Haven (DataLoch, Edinburgh, UK). Data describing patient demographics, presenting symptoms, previous medical conditions and revascularisation, medication at presentation, investigations, and laboratory measurements were extracted. This exploratory analysis was pre-specified in the trial protocol, however, due to its observational nature the statistical analysis plan was not reviewed by the trial steering committee.

### Procedures

In the High-STEACS trial, cardiac troponin testing was performed at presentation and was repeated 6 h or 12 h after the onset of symptoms at the discretion of the attending physician and in accordance with national guidelines.^13^ All patients had troponin measured using the investigational high-sensitivity assay (ARCHITECT STAT high-sensitive troponin I assay; Abbott Laboratories, Abbott Park, IL, USA) throughout the trial, but this was only used to guide clinical decisions during the implementation phase. Attending clinicians were masked to the results of the high-sensitivity assay during the validation phase when a contemporary assay was used to guide care. This assay has an inter-assay coefficient of variation of less than 10% at 4·7 ng/L,^14^ and a 99th centile upper reference limit of 34 ng/L in men and 16 ng/L in women.^14^

In the High-STEACS trial, all deaths and all diagnoses in patients with hs-cTnI concentrations above the 99th centile were adjudicated and diagnoses classified according to the third universal definition of myocardial infarction as previously described.^12^ In brief, two physicians independently reviewed all clinical information with discordant diagnoses resolved by a third reviewer. Type 1 myocardial infarction was defined as myocardial necrosis (any hs-cTnI concentration above the sex-specific 99th centile with a rise or fall in hs-cTnI concentration when serial testing was performed) in the context of a presentation with suspected acute coronary syndrome with symptoms or signs of myocardial ischemia on the electrocardiogram. Patients with myocardial necrosis, symptoms or signs of myocardial ischemia, and evidence of increased myocardial oxygen demand or decreased supply secondary to an alternative condition without evidence of acute atherothrombosis were defined as type 2 myocardial infarction. Type 4b myocardial infarction was defined where myocardial ischaemia and myocardial necrosis were associated with stent thrombosis documented at angiography. We used regional and national registries to ensure complete follow-up for the trial population.^12^

The primary outcome of this analysis was myocardial infarction (type 1 or type 4b) during the index visit. The key secondary outcomes were subsequent myocardial infarction (type 1 or type 4b) or cardiovascular death at 1 year, and all-cause death at one year.

For the current study, we derived the MI^3^ score using the high-sensitivity cardiac troponin I assay results. MI^3^ is an algorithm derived using the machine learning technique, gradient boosting. It computes a value of 0 to 100 for each patient using their age, sex, serial cardiac troponin concentrations, and the time interval between sampling, which corresponds to an individualised estimate of the likelihood of a diagnosis of type 1 or type 4b myocardial infarction.^11^

### Statistical analysis

Model discrimination was assessed by calculating the area under the receiver-operating-characteristic curve (AUROC) and model calibration was assessed by visual inspection of the calibration and precision-recall curve. Diagnostic performance was evaluated using the previously defined low-probability threshold (MI^3^ score of 1·6) and high-probability threshold (MI^3^ score of 49·7).^11^ These thresholds were defined in the cohort used to train the algorithm based on pre-specified performance criteria (sensitivity ≥99·0% and negative predictive value [NPV] ≥99·5% for low-probability, specificity ≥90·0% and positive predictive value [PPV] ≥75·0% for high-probability). We report the sensitivity, specificity, NPV, and PPV for these thresholds, along with 95% CI calculated using 1000 bootstrapped samples. Survival free from subsequent myocardial infarction or cardiovascular death at 1 year, or death from any cause at 1 year was determined in patients grouped according to their MI^3^ score (low-probability <1·6; intermediate-probability 1·6–49·6; high-probability ≥49·7). The event rates were compared using a χ^2^ test and a log-rank test. Subgroup analysis was performed by age (<65 years or ≥65 years), sex (male or female), primary symptom of chest pain, previous ischaemic heart disease, myocardial infarction, diabetes, and cerebrovascular disease and stratified by renal function (estimated glomerular filtration rate [eGFR] <60 ml/min or ≥60 ml/min) and the time from symptom onset to presentation (<3 h, 3–6 h, and >6 h). MI^3^ performance was also validated based on the time interval between blood sampling (<3 h, 3–6 h, and >6 h). In a sensitivity analysis, we evaluated diagnostic performance for a composite endpoint of type 1, type 4b, or type 2 myocardial infarction during the index hospital admission. All analyses were conducted using R (version 3.6.1).

### Role of the funding source

The funders of the study had no role in study design, data collection, data analysis, data interpretation, or writing of the report.

## Results

**Figure 1:**
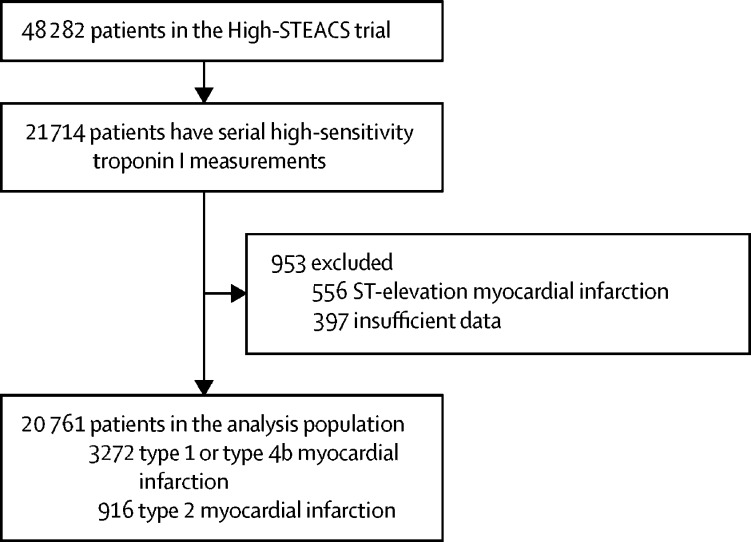
Flow diagram of the analysis population

Between June 10, 2013, and March 3, 2016, all patients with suspected acute coronary syndrome who met the eligibility criteria for the High-STEACS trial were identified. Of 48 282 patients in the trial, 20 761 patients (SD 16; 9597 [46%] women) enrolled in the validation and implementation phases of the trial were included in this analysis (figure 1). There were no differences (assessed visually between the full trial population and the analysis population) in sex distribution, presenting complaint, or laboratory markers, including cardiac troponin concentrations, but the analysis population was on average 3 years older than the trial population, and patients were more likely to have a previous history of ischaemic heart disease and to be established on preventative medication (table). In the analysis population, cardiac troponin concentrations were above the 99th centile in 5788 (27·9%) of 20 761 patients at presentation or on serial testing. The adjudicated diagnosis was type 1 or type 4b myocardial infarction in 3272 (15·8%) patients and type 2 myocardial infarction in 916 (4·4%) patients.

**Table:** Baseline characteristics of the analysis population stratified by MI^3^ probability score

		**High-STEACS trial**		**MI^3^ group**		
		All participants	Analysis population	Low probability	Intermediate probability	High probability
Number of participants		48 282	20 761	12 983/20 761 (62·5%)	4817/20 761 (23·%)	2961/20 761 (14·3%)
Mean age, years		61 (17)	64 (16)	59 (15)	72 (14)	69 (14)
Sex						
	Female	22 562 (46·7%)	9597 (46·2%)	6241 (48·1%)	2225 (46·2%)	1131 (38·2%)
	Male	25 720 (53·3%)	11 164 (53·8%)	6742 (51·9%)	2592 (53·8%)	1830 (61·8%)
Presenting complaint*						
	Chest pain	34 540 (81·0%)	15 878 (85·9%)	10 430 (91·0%)	3291 (76·3%)	2157 (79·3%)
	Dyspnoea	2175 (5·1%)	709 (3·8%)	171 (1·5%)	326 (7·6%)	212 (7·8%)
	Palpitation	1269 (3·0%)	336 (1·8%)	164 (1·4%)	131 (3·0%)	41 (1·5%)
	Syncope	2495 (5·8%)	868 (4·7%)	393 (3·4%)	335 (7·8%)	140 (5·1%)
	Other	2188 (5·1%)	706 (3·8%)	309 (2·7%)	227 (5·3%)	170 (6·3%)
Previous medical conditions						
	Myocardial infarction	4214 (8·7%)	2504 (12·1%)	1317 (10·1%)	777 (16·1%)	413 (13·9%)
	Ischaemic heart disease	11 912 (24·7%)	6746 (32·5%)	3666 (28·2%)	2126 (44·1%)	954 (32·2%)
	Cerebrovascular disease	2949 (6·1%)	1414 (6·8%)	624 (4·8%)	564 (11·7%)	226 (7·6%)
	Diabetes	3518 (7·3%)	1960 (9·4%)	781 (6·0%)	687 (14·3%)	492 (16·6%)
Previous revascularisation						
	Percutaneous coronary intervention	3682 (7·6%)	2229 (10·7%)	1330 (10·2%)	597 (12·4%)	302 (10·2%)
	Coronary artery bypass grafting	782 (1·6%)	446 (2·2%)	216 (1·7%)	163 (3·4%)	67 (2·3%)
Medications at presentation						
	Aspirin	13 163 (27·3%)	7021 (33·8%)	3934 (30·3%)	1993 (41·4%)	1094 (36·9%)
	Dual anti-platelet therapy†	1605 (3·3%)	965 (4·7%)	515 (4·0%)	298 (6·2%)	152 (5·1%)
	Statin	19 366 (40·1%)	9957 (48·0%)	5609 (43·2%)	2819 (58·5%)	1529 (51·6%)
	ACE inhibitor or ARB	15 618 (32·3%)	7948 (38·3%)	4390 (33·8%)	2292 (47·6%)	1266 (42·8%)
	β blocker	13 173 (27·3%)	6804 (32·8%)	3844 (29·6%)	1898 (39·4%)	1062 (35·9%)
	Oral anticoagulant‡	3253 (6·7%)	1529 (7·4%)	663 (5·1%)	650 (13·5%)	216 (7·3%)
Haematology and clinical chemistry measurements						
	Mean haemoglobin, g/L	136 (22)	135 (21)	137 (20)	130 (24)	134 (24)
	Mean estimated glomerular filtration, mL/min	54 (13)	54 (12)	57 (9)	50 (14)	49 (15)
	Median peak high-sensitivity cardiac troponin I, ng/L	4 (2–16)	5 (2–18)	2 (1–4)	19 (12–41)	133 (40–574)

Data are mean (SD), median (IQR), n/N (%), or n (%). ACE=angiotensin converting enzyme. ARB=angiotensin receptor blockers. MI^3^=myocardial-ischaemic-injury-index.

* A presenting symptom was missing in 5615 (12%) from all participants (n=48 282) and 2264 (11%) from the analysis population (n=20 761), hence the difference in the proportions.

† Two medications from aspirin, clopidogrel, prasugrel, or ticagrelor.

‡ Includes warfarin or novel oral anticoagulants.

**Figure 2:**
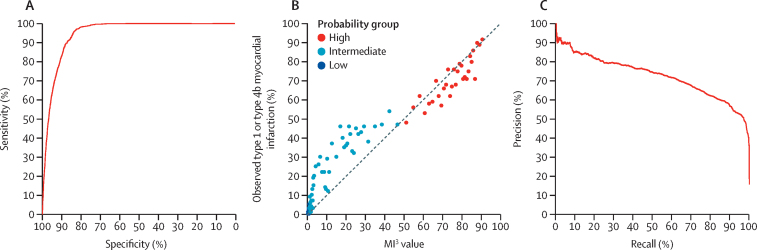
Overall diagnostic performance of the MI^3^ algorithm (A) Receiver-operating-characteristic curve illustrating discrimination of the MI^3^ algorithm for type 1 or type 4b myocardial infarction. (B) Calibration of the MI^3^ algorithm with the observed proportion of patients with type 1 or type 4b myocardial infarction. The dashed line represents perfect calibration. Each point represents 100 patients. (C) Precision-recall curve illustrating discrimination of the MI^3^ algorithm for type 1 or type 4b myocardial infarction. MI^3^=myocardial-ischaemic-injury-index.

**Figure 3:**
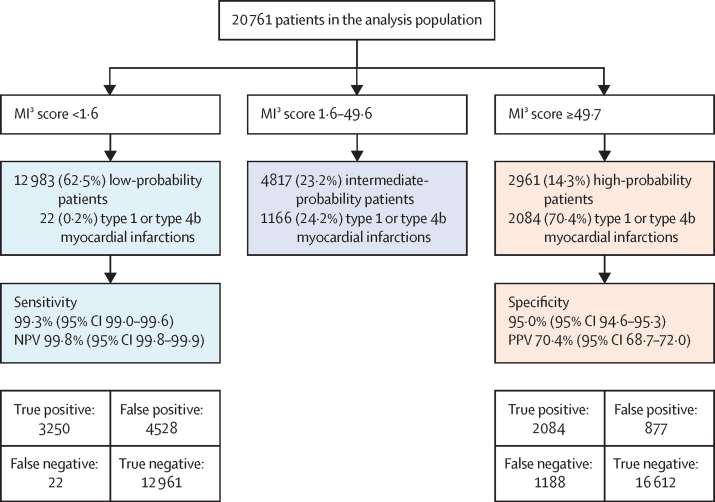
Performance of MI^3^ at pre-defined thresholds MI^3^=myocardial-ischaemic-injury-index. NPV=negative predictive value. PPV=positive predictive value.

The MI^3^ algorithm performed well overall with an AUROC of 0·949 (95% CI 0·946–0·952) discriminating between those with and without type 1 or type 4b myocardial infarction. Discrimination was similar in patients evaluated during the validation and implementation phases of the trial (AUROC 0·949 [95% CI 0·944–0·954] *vs* 0·948 [0·945–0·952]). However, calibration was not good in patients with intermediate MI^3^ scores. MI^3^ scores between 10 and 40 underestimated the observed risk, and scores between 65 and 86 overestimated the observed risk (figure 2). MI^3^ identified 12 983 (62·5%) of 20 761 patients as low-probability for type 1 or type 4b myocardial infarction at the pre-specified threshold (MI^3^ score <1·6), with a sensitivity of 99·3% (95% CI 99·0–99·6) and NPV of 99·8% (95% CI 99·8–99·9; figure 3; appendix p 11). MI^3^ identified 2961 (14·3%) of 20 761 patients as high-probability at the pre-specified threshold (MI^3^ score ≥49·7), with a specificity of 95·0% (95% CI 94·6–95·3) and PPV of 70·4% (95% CI 68·7–72·0; figure 3; appendix p 11).

The AUROC differed when stratifying patients by subgroups and was higher in those aged <65 years, in males, those presenting with a primary symptom of chest pain, eGFR ≥60 ml/min, no previous ischaemic heart disease, myocardial infarction, diabetes, and cerebrovascular disease, but there was no difference when stratifying by time from symptom onset to presentation (appendix p 3). Among subgroups there was no heterogeneity (overlap of 95%CIs) in sensitivity or NPV for the low-probability threshold (appendix pp 4–5), and in some subgroups, there was significant heterogeneity in the specificity and PPV for the high-probability threshold (appendix pp 6–7). In particular, the PPV for the high-probability threshold was higher in patients with a primary presenting symptom of chest pain than patients with other presenting symptoms (80·3% [95% CI 78·6–81·9%] *vs* 34·6% [30·5–38·8%]).

MI^3^ performed similarly in patients who had serial measurements within 3 h (2439 [11·8%], AUROC 0·972 [95% CI 0·966–0·979]) or between 3 and 6 h (4098 [19·7%], AUROC 0·968 [95% CI 0·963–0·973]). The MI^3^ algorithm also performed well in patients with serial measurements more than 6 h apart, but discrimination was lower in this group (14 224 [68·5%], AUROC 0·939 [95% CI 0·935–0·942]; appendix p 8).

In the analysis population, 4188 (20·2%) patients had an adjudicated diagnosis of type 1, type 4b, or type 2 myocardial infarction. Discrimination was improved for the composite outcome of type 1, type 4b, or type 2 myocardial infarction (AUROC 0·963 [95% CI 0·960–0·965]; appendix p 9) compared with type 1 or type 4b myocardial infarction alone (AUROC 0·949 [95% CI 0·946–0·952]); however, calibration was not so good. The performance of the high-probability threshold was improved (specificity 97·1% [95% CI 96·8–97·3], PPV 83·7% [95% CI 82·3–85·0]) identifying 2961 (14·3%) of 20 761 patients, and the low-probability threshold identified 12 983 (62·5%) of 20 761 patients with no difference in performance (sensitivity 99·3% [95% CI 99·0–99·5], NPV 99·8% [95% CI 99·7–99·8]; appendix p 10).

**Figure 4:**
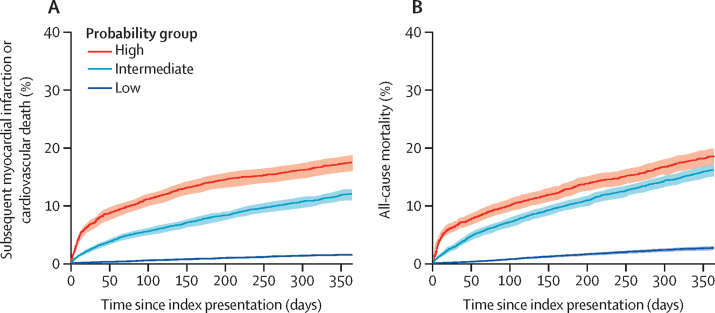
Cumulative incidence of myocardial infarction or cardiovascular death over 1 year (A) and death from any cause stratified by MI^3^ score (B) Low probability was an MI^3^ score of less than 1·6, intermediate probability was an MI^3^ score of 1·6 to 49·6, and high probability was an MI^3^ score of 49·7 or more. Log-rank between groups for both endpoints, p<0·0001.

In the analysis population, 1300 (6·3%) patients had either a subsequent myocardial infarction or cardiovascular death at 1 year. Patients identified by MI^3^ as high-probability of index myocardial infarction were more likely to have a subsequent myocardial infarction or cardiovascular death than patients identified as low-probability (520 [17·6%] of 2961] *vs* 197 [1·5%] of 12 983, p<0·0001; figure 4). Death from any cause occurred in 1671 (8%) patients, and MI^3^ was also a good predictor of all-cause mortality (low-probability, 344 [2·6%] of 12 983; intermediate-probability, 776 [16·1%] of 4817; high-probability, 551 [18·6%] of 2961).

## Discussion

We validated the MI^3^ machine learning algorithm for the diagnosis of myocardial infarction in a large cohort of consecutive patients undergoing serial cardiac troponin measurement for suspected acute coronary syndrome in a multi-centre randomised trial. We make several observations that could inform its application in clinical practice. First, MI^3^ discriminated for type 1 or type 4b myocardial infarction in a patient population that reflects routine clinical practice. However, calibration was not good in patients with intermediate MI^3^ scores. Second, at the prespecified score thresholds, sensitivity and NPV were consistent across patient subgroups; however, specificity and PPV varied substantially. Third, MI^3^ provided insights beyond the index presentation, identifying patients at risk of future adverse cardiovascular events. Patients identified as high-probability by the algorithm had a ten-times higher rate of subsequent myocardial infarction or cardiovascular death at 1 year than patients who were classified as low-probability (17·6% *vs* 1·5%).

Our study population had several characteristics that enabled a robust evaluation of the MI^3^ algorithm. Compared with the pooled cohort used in the initial validation of MI^3^,^11^ our external validation population is almost three times larger and consists of consecutive patients, improving the generalisability of our findings. The mean age in our study population is more than 5 years older, with a more balanced sex distribution and a higher prevalence of comorbidities than the populations used to train and test the model. Although the prevalence of type 1 myocardial infarction in the model training set was 13·4% and 10·6% in the testing set, in the external validation set the prevalence was 15·8%. Although the prevalence was slightly higher in the external validation set, the distribution of MI^3^ scores across the low-risk, intermediate-risk, and high-risk groups was similar. These features are likely to have resulted in a patient population in which there is more diagnostic complexity that is more reflective of clinical practice. Although the rule-in performance was more heterogeneous across patient subgroups, MI^3^ had an excellent rule-out performance across the study population (PPV *vs* NPV). This observation is perhaps unsurprising given that high-sensitivity cardiac troponin, a key variable in this algorithm, is integral to the diagnosis of myocardial infarction and is known to be influenced by both age and sex.^15^, ^16^

Given that the probability of type 1 myocardial infarction and cardiac troponin concentrations can differ substantially in different patient subgroups^17^ it is perhaps intuitive that a diagnostic algorithm that combines cardiac troponin and clinical parameters has a good diagnostic performance. Indeed, there have been numerous statistical models developed to aid in the diagnosis and prognostication of acute cardiovascular conditions, including type 1 myocardial infarction.^18^, ^19^, ^20^, ^21^ However, very few have been successfully implemented into clinical practice due to barriers such as the number and complexity of the variables that are required in the models and the lack of adequate validation to have sufficient confidence in the diagnostic performance.^22^, ^23^, ^24^ We have not compared gradient boosting with other models or forms of statistical modelling, nor have we evaluated whether discrimination or calibration can be improved by including additional parameters. The objectivity and simplicity of the variables used by MI^3^ are perhaps the algorithm's most important strength. The three variables used in this algorithm (age, sex, and troponin) are objective and consistently obtained, with high reproducibility and accuracy, in a busy clinical setting. Furthermore, the initial validation of this algorithm was performed in an international multicentre patient population, and its diagnostic performance has now been validated in a large consecutive patient population that reflects clinical practice.

Our data further supports the potential clinical application of decision support tools that incorporate key patient factors in the interpretation of cardiac biomarkers. High-sensitivity cardiac troponin is well known to vary substantially according to various patient factors such as age and sex, however it is difficult to account for the complex relationships between these variables using a threshold-based approach. Moreover, the data demonstrate that no single threshold provides optimal sensitivity and specificity, and therefore we propose the use of separate MI^3^ thresholds to identify patients who are at low-risk risk of myocardial infarction that optimise sensitivity or NPV and patients who are at high-risk of myocardial infarction that optimise specificity or PPV. Many institutions worldwide have not yet implemented the sex-specific 99th centile thresholds recommended by the universal definition of myocardial infarction.^25^, ^26^ MI^3^ could help as it enables more accurate and individualised clinical decisions by accounting for the patient's age and sex in a manner that can be easily interpreted. Furthermore, the ability to include serial troponin concentrations at flexible time points reduces the potential of misinterpretation compared with an approach that recommends the use of fixed absolute changes in cardiac troponin at specific time-points.^27^, ^28^ In our cohort, MI^3^ was able to rule out myocardial infarction in the majority of patients with a high NPV irrespective of when testing was performed, while identifying 14·3% of patients with a high probability of myocardial infarction.

The application of this algorithm in practice would represent a substantial change in the approach to the assessment of patients with suspected acute coronary syndrome. Our current practice is based exclusively on the use of single or multiple cardiac troponin thresholds with serial testing performed at fixed timepoints. By using cardiac troponin as a continuous measure and incorporating rate of change rather than an absolute change in troponin concentration, MI^3^ might be more flexible and easier to implement in busy emergency departments. To our knowledge no similar algorithms are available and none report the likelihood of myocardial infarction for individual patients or associated diagnostic metrics to guide clinical decision making. Although we have validated the performance of the algorithm in triaging patients as low, intermediate, or high risk, in practice we would anticipate that clinical decisions are guided by individualised estimates of the diagnostic likelihood. Further studies are required to evaluate whether care guided by these estimates, and the provision of diagnostic metrics, changes clinical decision making, or the use of subsequent cardiac testing in practice.

Although the training and testing of this algorithm has been published previously,^11^ this is the first time that MI^3^ has been validated in a consecutive patient population that reflects the way it could be applied in clinical practice. This is an essential step in understanding how the algorithm will perform in practice whereby troponin testing is guided by clinical need rather than by a research protocol. The lack of external validation and evaluations of algorithm performance in routine care is one of the main reasons that few machine learning algorithms are used in practice today. Furthermore, in addition to validating the diagnostic performance of MI^3^, we provide, for the first-time, data on outcomes following discharge from hospital. The association with adverse cardiovascular outcomes at 1 year is reassuring and suggests that the algorithm is appropriately risk stratifying patients who are likely to benefit most from further diagnostic testing and treatment beyond the index visit.

Although MI^3^ had a good overall diagnostic performance in our cohort, there are several limitations and aspects that can potentially be improved. First, we observed that MI^3^ was not well calibrated in patients with intermediate scores. This group of patients are the most challenging to diagnose in clinical practice because they often have small elevations in cardiac troponin, which might be due to conditions other than myocardial infarction. One of the advantages of using a machine learning algorithm over other pathways is that further training is possible, which might be required to improve calibration for this group in different health-care settings. Alternatively, the use of additional features to refine the estimates of probability in this group could be explored. Second, although performance of the low-probability threshold was consistent across important patient subgroups, we observed heterogeneity in the PPV of the high-probability threshold, particularly when stratified according to the primary presenting symptom. This finding is consistent with our previous research^29^, ^30^ and probably reflects the greater prevalence of non-ischaemic myocardial injury and type 2 myocardial infarction in our consecutive patient population as compared with the cohorts used to train the algorithm whereby some patient selection was inevitable. It is possible that an algorithm that incorporates other clinical features might perform more consistently across these subgroups when identifying patients at high probability of type 1 myocardial infarction. Third, we used serial cardiac troponin measurements for both the rule-in and rule-out of myocardial infarction. Algorithms that can risk stratify patients using only cardiac troponin concentrations at presentation could be developed and might further improve efficiency. Finally, although MI^3^ had good performance for the prediction of type 1 or type 2 myocardial infarction, it was not developed to distinguish between the two. Future algorithms to diagnose and differentiate between type 1 and type 2 myocardial infarction would be useful given the diagnostic challenge of doing so in clinical practice and that the treatment for these conditions differs.

We also acknowledge several limitations in our study design. In most patients in our cohort, serial cardiac troponin measurements were performed 6 h apart, which is longer than recommended by current international guidelines.^27^ However, in our subgroup analysis stratified by time of serial sampling, the diagnostic performance of MI^3^ remained good regardless of the time interval between serial troponin measurements. Although MI^3^ includes sex as a parameter in the model discrimination was not as good in women compared to men. This finding probably reflects differences in the use of sex-specific and uniform thresholds to diagnose myocardial infarction between the data sets used to train and to validate the algorithm.^11^ In the High-STEACS trial, sex-specific thresholds were used in practice and to adjudicate the diagnosis of myocardial infarction in line with the recommendations of the universal definition of myocardial infarction.^16^ These recommendations were not consistently applied in the populations used to train the algorithm. Performance could be improved with additional training of the algorithm in health-care settings that use sex-specific diagnostic thresholds in practice. A further limitation is that we did not have access to data on ethnicity to evaluate whether diagnostic performance also varied across ethnic groups. Finally, although our analysis demonstrated that the low number of adverse cardiovascular outcomes at 1 year in patients classified as low-probability by MI^3^ was reassuring, future studies evaluating outcomes after MI^3^ is implemented are needed to confirm the safety of this algorithm in clinical practice.

In consecutive patients undergoing serial cardiac troponin measurement for suspected acute coronary syndrome, the MI^3^ machine learning algorithm can accurately estimate the likelihood of myocardial infarction and predict subsequent adverse cardiovascular events. The model could improve the diagnostic pathways for myocardial infarction by accurately identifying patients at high risk to be targeted for prompt individualised treatment, and by allowing early discharge in patients at low risk.

## Data sharing

The High-STEACS trial makes use of several routine electronic health-care data sources that are linked, de-identified, and held in DataLoch Safe Haven, which is accessible by approved individuals who have undertaken the necessary governance training. Summary aggregate level data and analysis code for this study can be made available upon request to the corresponding author. The algorithm is proprietary and subject to a patent application but we can share it for research purposes with a data sharing agreement upon request to the corresponding author.

## Declaration of interests

NLM has received honoraria or consultancy from Abbott Diagnostics, Roche Diagnostics, Siemens Healthineers, and LumiraDx. KKL has received honoraria from Abbott Diagnostics. ASVS's institution (the University of Edinburgh) has received speaker fees from Abbott Diagnostics. JWP has undertaken consultancy for Abbott Diagnostics. MPT has received consulting fees, honoraria, or payment from Abbott, Roche, and Siemens; funding for clinical research from Radiometer; and participated on a data safety monitoring board or an advisory board for Abbott, Radiometer, Roche, and Siemens. All other authors declare no competing interests.

## References

[1] Sanchis-Gomar F, Perez-Quilis C, Leischik R, Lucia A (2016). Epidemiology of coronary heart disease and acute coronary syndrome. Ann Transl Med.

[2] Collet JP, Thiele H, Barbato E (2020). The ‘Ten Commandments’ for the 2020 ESC Guidelines for the management of acute coronary syndromes in patients presenting without persistent ST-segment elevation. Eur Heart J.

[3] Goodacre S, Cross E, Arnold J, Angelini K, Capewell S, Nicholl J (2005). The health care burden of acute chest pain. Heart.

[4] Babuin L, Jaffe AS (2005). Troponin: the biomarker of choice for the detection of cardiac injury. CMAJ.

[5] Body R, Carley S, McDowell G (2011). Rapid exclusion of acute myocardial infarction in patients with undetectable troponin using a high-sensitivity assay. J Am Coll Cardiol.

[6] Body R, Carlton E, Sperrin M (2017). Troponin-only Manchester Acute Coronary Syndromes (T-MACS) decision aid: single biomarker re-derivation and external validation in three cohorts. Emerg Med J.

[7] Reichlin T, Schindler C, Drexler B (2012). One-hour rule-out and rule-in of acute myocardial infarction using high-sensitivity cardiac troponin T. Arch Intern Med.

[8] Than M, Flaws D, Sanders S (2014). Development and validation of the Emergency Department Assessment of Chest pain Score and 2 h accelerated diagnostic protocol. Emerg Med Australas.

[9] Chapman AR, Anand A, Boeddinghaus J (2017). Comparison of the efficacy and safety of early rule-out pathways for acute myocardial infarction. Circulation.

[10] Thygesen K, Alpert JS, Jaffe AS, Simoons ML, Chaitman BR, White HD (2012). Third universal definition of myocardial infarction. Circulation.

[11] Than MP, Pickering JW, Sandoval Y (2019). Machine learning to predict the likelihood of acute myocardial infarction. Circulation.

[12] Shah ASV, Anand A, Strachan FE (2018). High-sensitivity troponin in the evaluation of patients with suspected acute coronary syndrome: a stepped-wedge, cluster-randomised controlled trial. Lancet.

[13] Scottish Intercollegiate Guidelines Network (2013). Acute coronary syndromes. https://www.sign.ac.uk/sign-148-acute-coronary-syndrome.

[14] Shah AS, Anand A, Sandoval Y (2015). High-sensitivity cardiac troponin I at presentation in patients with suspected acute coronary syndrome: a cohort study. Lancet.

[15] Mueller-Hennessen M, Lindahl B, Giannitsis E (2016). Diagnostic and prognostic implications using age- and gender-specific cut-offs for high-sensitivity cardiac troponin T — sub-analysis from the TRAPID-AMI study. Int J Cardiol.

[16] Lee KK, Ferry AV, Anand A (2019). Sex-specific thresholds of high-sensitivity troponin in patients with suspected acute coronary syndrome. J Am Coll Cardiol.

[17] Shah ASV, Sandoval Y, Noaman A (2017). Patient selection for high sensitivity cardiac troponin testing and diagnosis of myocardial infarction: prospective cohort study. BMJ.

[18] Eggers KM, Ellenius J, Dellborg M (2007). Artificial neural network algorithms for early diagnosis of acute myocardial infarction and prediction of infarct size in chest pain patients. Int J Cardiol.

[19] Green M, Björk J, Forberg J, Ekelund U, Edenbrandt L, Ohlsson M (2006). Comparison between neural networks and multiple logistic regression to predict acute coronary syndrome in the emergency room. Artif Intell Med.

[20] Rahimi K, Bennett D, Conrad N (2014). Risk prediction in patients with heart failure: a systematic review and analysis. JACC Heart Fail.

[21] Wessler BS, Lai Yh L, Kramer W (2015). Clinical prediction models for cardiovascular disease: tufts predictive analytics and comparative effectiveness clinical prediction model database. Circ Cardiovasc Qual Outcomes.

[22] Califf RM, Pencina MJ (2013). Predictive models in heart failure: who cares?. Circ Heart Fail.

[23] Damen JA, Hooft L, Schuit E (2016). Prediction models for cardiovascular disease risk in the general population: systematic review. BMJ.

[24] Siontis GC, Tzoulaki I, Castaldi PJ, Ioannidis JP (2015). External validation of new risk prediction models is infrequent and reveals worse prognostic discrimination. J Clin Epidemiol.

[25] Thygesen K, Alpert JS, Jaffe AS (2018). Fourth universal definition of myocardial infarction (2018). Circulation.

[26] Anand A, Shah ASV, Beshiri A, Jaffe AS, Mills NL (2019). Global adoption of high-sensitivity cardiac troponins and the universal definition of myocardial infarction. Clin Chem.

[27] Roffi M, Patrono C, Collet JP (2016). 2015 ESC Guidelines for the management of acute coronary syndromes in patients presenting without persistent ST-segment elevation: task force for the management of acute coronary syndromes in patients presenting without persistent ST-segment elevation of the European Society of Cardiology (ESC). Eur Heart J.

[28] Neumann JT, Twerenbold R, Ojeda F (2019). Application of high-sensitivity troponin in suspected myocardial infarction. N Engl J Med.

[29] Lee KK, Noaman A, Vaswani A (2019). Prevalence, determinants, and clinical associations of high-sensitivity cardiac troponin in patients attending emergency departments. Am J Med.

[30] Wereski R, Kimenai DM, Taggart C (2021). Cardiac troponin thresholds and kinetics to differentiate myocardial injury and myocardial infarction. Circulation.

