# Supplementary material for: Validation of the myocardial-ischaemic-injury-index machine learning algorithm to guide the diagnosis of myocardial infarction in a heterogenous population: a prespecified exploratory analysis
Source: Lancet Digit Health. 2022 Apr 20;4(5):e300–8. doi: 10.1016/S2589-7500(22)00025-5 (PMC9052331; doi:10.1016/S2589-7500(22)00025-5)
Supplement: Supplementary appendix [file mmc1.pdf]

THE LANCET
Digital Health

Supplementary appendix

This appendix formed part of the original submission and has been peer reviewed. We post it as supplied by the authors.

Supplement to: Doudesis D, Lee KK, Yang J, et al. Validation of the myocardial-ischaemic-injury-index machine learning algorithm to guide the diagnosis of myocardial infarction in a heterogenous population: a prespecified exploratory analysis. *Lancet Digit Health* 2022; **4:** e300–08.

Supplement

SUPPLEMENTAL FIGURE LEGENDS AND TABLES

Supplemental Figure S1. Forest plot of the discrimination (AUC) across patient subgroups. AUC = area under curve.

Supplemental Figure S2. Forest plot of the sensitivity across patient subgroups. TP = true positive, FN = false negative. The vertical line represents the performance target.

Supplemental Figure S3. Forest plot of the negative predictive value across patient subgroups. NPV = negative predictive value, TN = true negative, FN = false negative. The vertical line represents the performance target.

Supplemental Figure S4. Forest plot of the specificity across patient subgroups. TN = true negative, FP = false positive. The vertical line represents the performance target.

Supplemental Figure S5. Forest plot of the positive predictive value across patient subgroups. PPV = positive predictive value, TP = true positive, FP = false positive. The vertical line represents the performance target.

Supplemental Figure S6. Receiver-operating-characteristic (ROC) curve illustrating discrimination of the MI^3 algorithm for type 1 or type 4b myocardial infarction, stratified by the time difference between blood samples.

Supplemental Figure S7.

(a) Receiver-operating-characteristic (ROC) curve illustrating discrimination of the MI^3 algorithm for type 1, type 4b or type 2 myocardial infarction.

(b) Calibration of the MI^3 algorithm with the observed proportion of patients with type 1, type 4b or type 2 myocardial infarction. The dashed line represents perfect calibration. Each point represents 100 patients.

(c) Precision-recall curve illustrating discrimination of the MI^3 algorithm for type 1, type 4b or type 2 myocardial infarction.

Supplemental Figure S8. Performance of MI^3 at example thresholds. Secondary outcome: type 1, type 4b or type 2 myocardial infarction.
TP = true positive, FP = false positive, FN = false negative, TN = true negative, NPV = negative predictive value, PPV = positive predictive value.

Supplemental Table S1. Performance of MI^3 at example thresholds. Primary outcome: type 1 or type 4b myocardial infarction.
TP = true positive, FP = false positive, FN = false negative, TN = true negative, NPV = negative predictive value, PPV = positive predictive value.

Supplemental Table S2. Performance of MI^3 on the testing set compared to the external validation set. NPV = negative predictive value, PPV = positive predictive value.

Supplemental Figure S1.

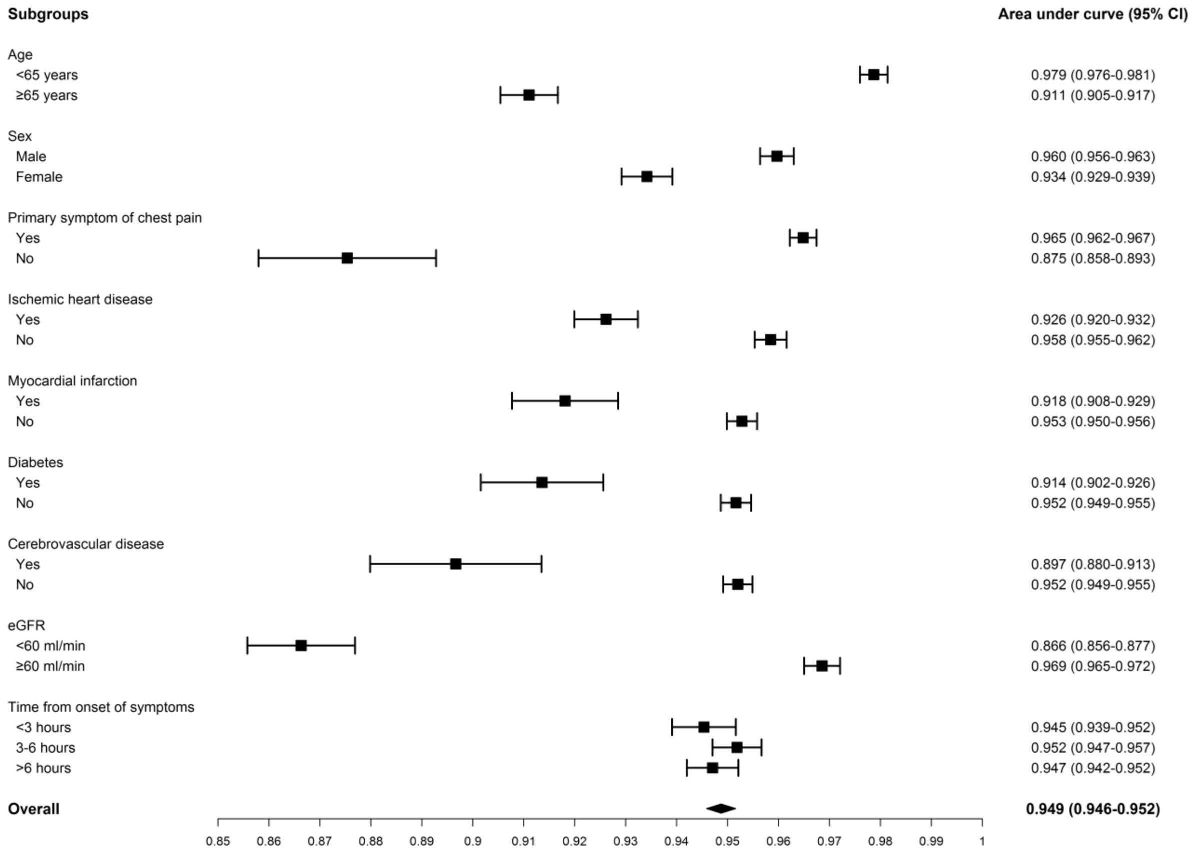

Supplemental Figure S2.

Subgroups	True positive	False negative	Sensitivity (95% CI)
Age			
<65 years	1263	7	99.4 (99.0-99.8)
≥65 years	1987	15	99.2 (98.8-99.6)
Sex			
Male	1875	4	99.8 (99.6-99.9)
Female	1375	18	98.7 (98.1-99.2)
Primary symptom of chest pain			
Yes	2702	19	99.3 (99.0-99.6)
No	276	0	100.0 (100.0-100.0)
Ischemic heart disease			
Yes	1181	10	99.1 (98.6-99.6)
No	2069	12	99.4 (99.1-99.7)
Myocardial infarction			
Yes	531	3	99.5 (98.7-100.0)
No	2719	19	99.3 (99.0-99.6)
Diabetes			
Yes	578	2	99.7 (99.2-100.0)
No	2672	20	99.2 (98.9-99.6)
Cerebrovascular disease			
Yes	255	2	99.2 (98.0-100.0)
No	2995	20	99.3 (99.0-99.6)
eGFR			
<60 ml/min	1019	5	99.5 (99.0-99.9)
≥60 ml/min	1136	7	99.4 (98.9-99.7)
Time from onset of symptoms			
<3 hours	747	7	99.1 (98.3-99.6)
3-6 hours	1068	7	99.4 (98.9-99.8)
>6 hours	1163	5	99.6 (99.2-99.9)
**Overall**	**3250**	**22**	**99.3 (99.0-99.6)**

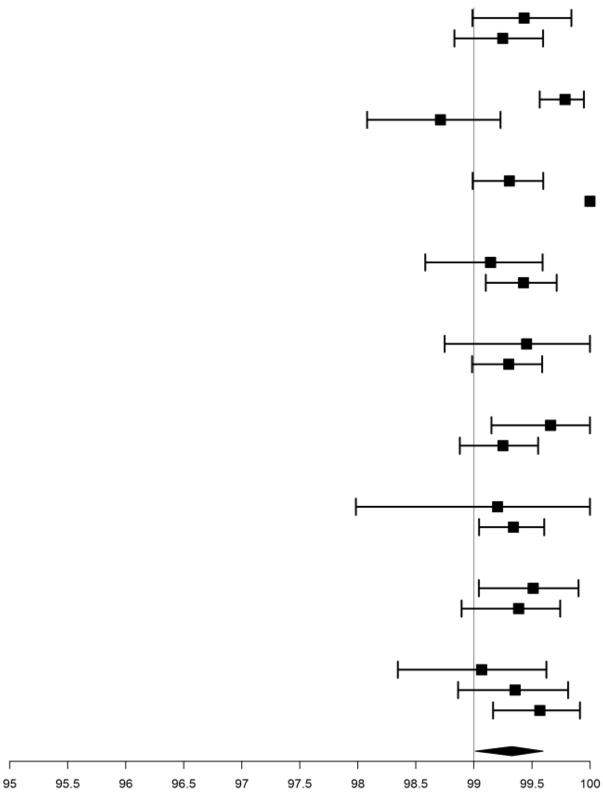

Supplemental Figure S3.

Subgroups	True negative	False negative	Negative predictive value (95% CI)
Age			
<65 years	8180	7	99.9 (99.8-100.0)
≥65 years	4781	15	99.7 (99.5-99.8)
Sex			
Male	6738	4	99.9 (99.9-100.0)
Female	6223	18	99.7 (99.6-99.8)
Primary symptom of chest pain			
Yes	10411	19	99.8 (99.7-99.9)
No	1037	0	100.0 (100.0-100.0)
Ischemic heart disease			
Yes	3656	10	99.7 (99.5-99.9)
No	9305	12	99.9 (99.8-99.9)
Myocardial infarction			
Yes	1314	3	99.8 (99.5-100.0)
No	11647	19	99.8 (99.8-99.9)
Diabetes			
Yes	779	2	99.7 (99.4-100.0)
No	12182	20	99.8 (99.8-99.9)
Cerebrovascular disease			
Yes	622	2	99.7 (99.2-100.0)
No	12339	20	99.8 (99.8-99.9)
eGFR			
<60 ml/min	1617	5	99.7 (99.4-99.9)
≥60 ml/min	6676	7	99.9 (99.8-100.0)
Time from onset of symptoms			
<3 hours	2818	7	99.8 (99.6-99.9)
3-6 hours	4194	7	99.8 (99.7-100.0)
>6 hours	4436	5	99.9 (99.8-100.0)
**Overall**	**12961**	**22**	**99.8 (99.8-99.9)**

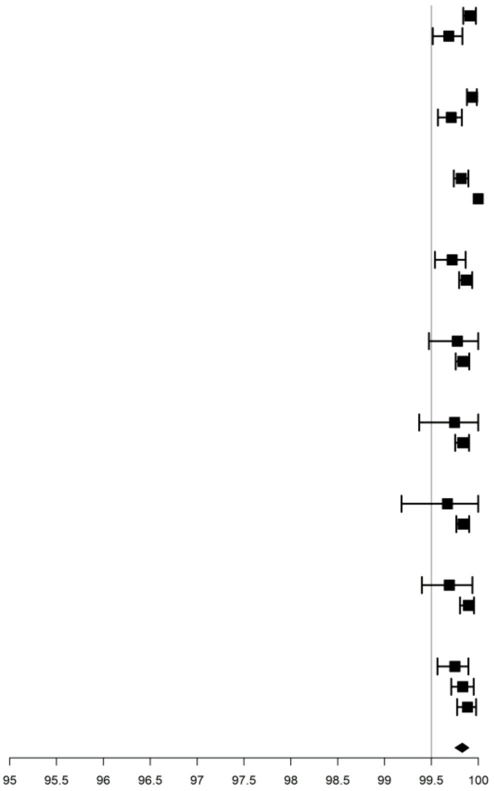

Supplemental Figure S4.

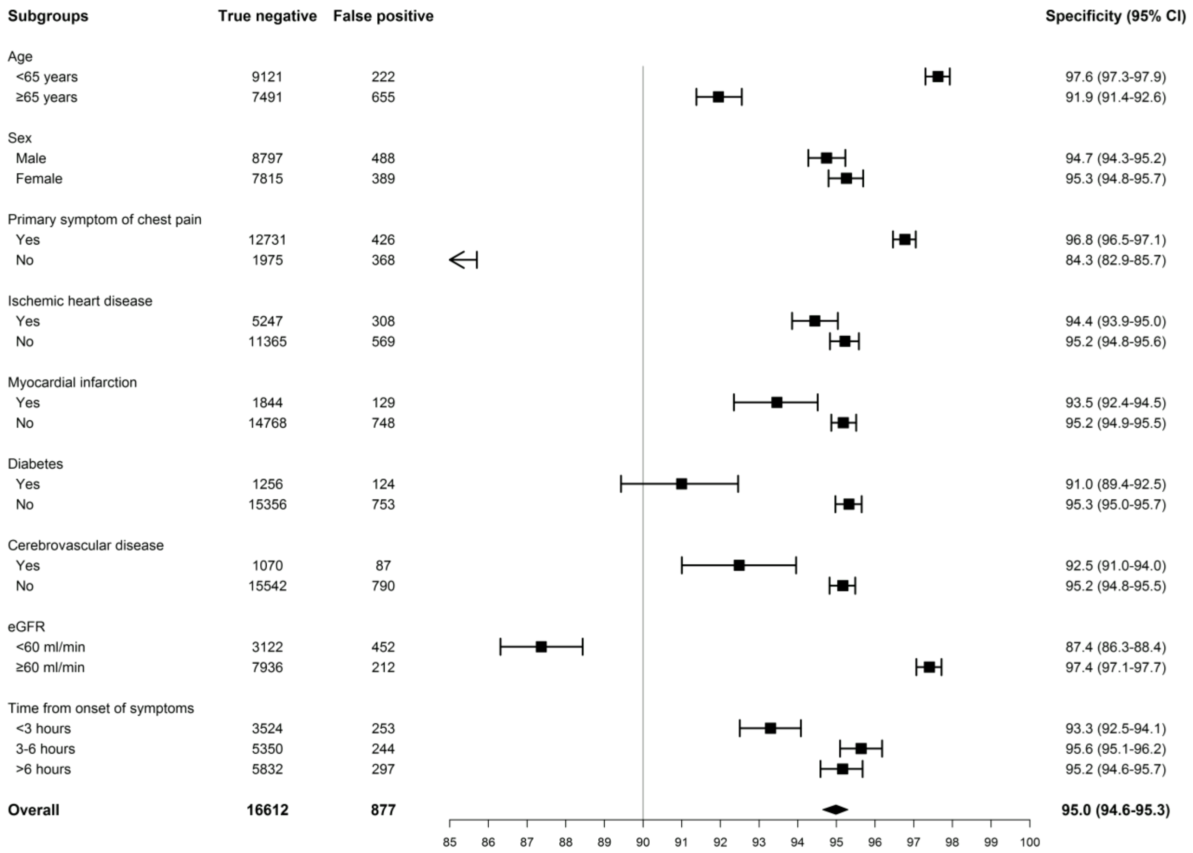

Supplemental Figure S5.

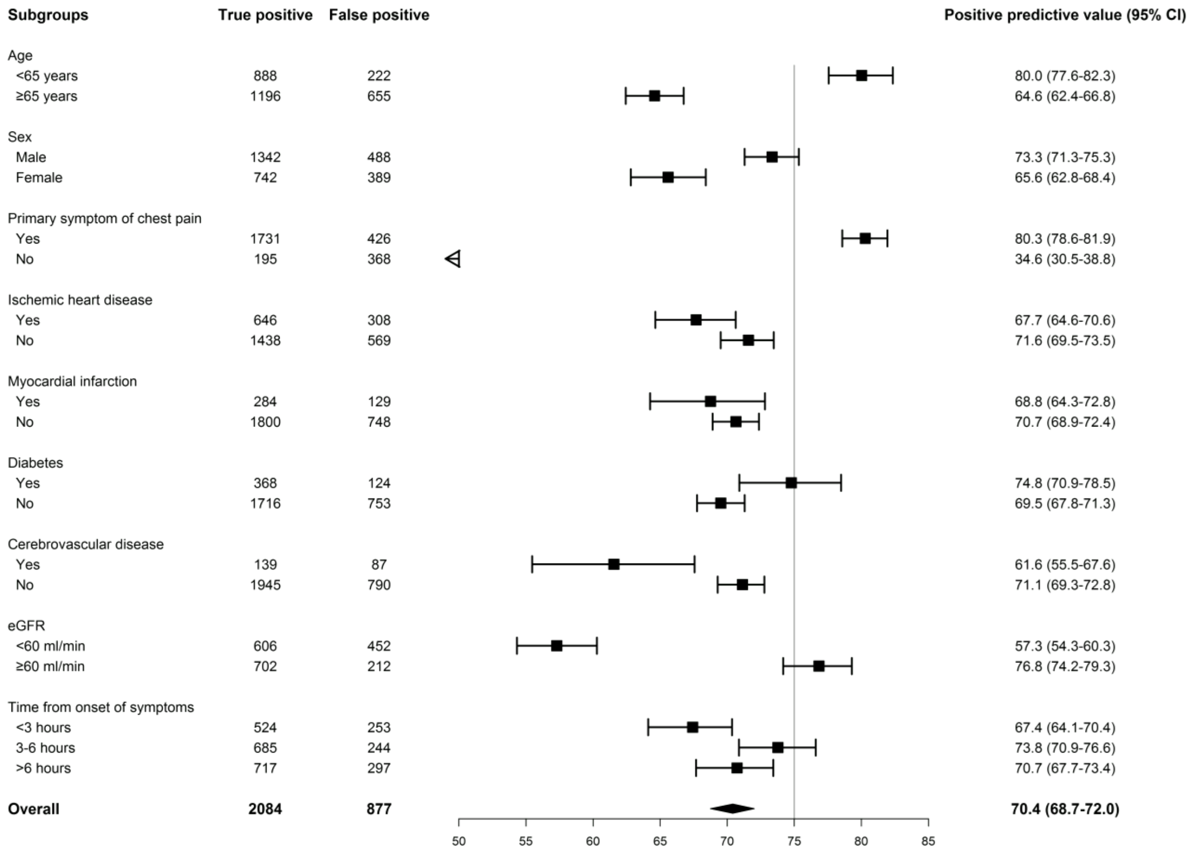

Supplemental Figure S6.

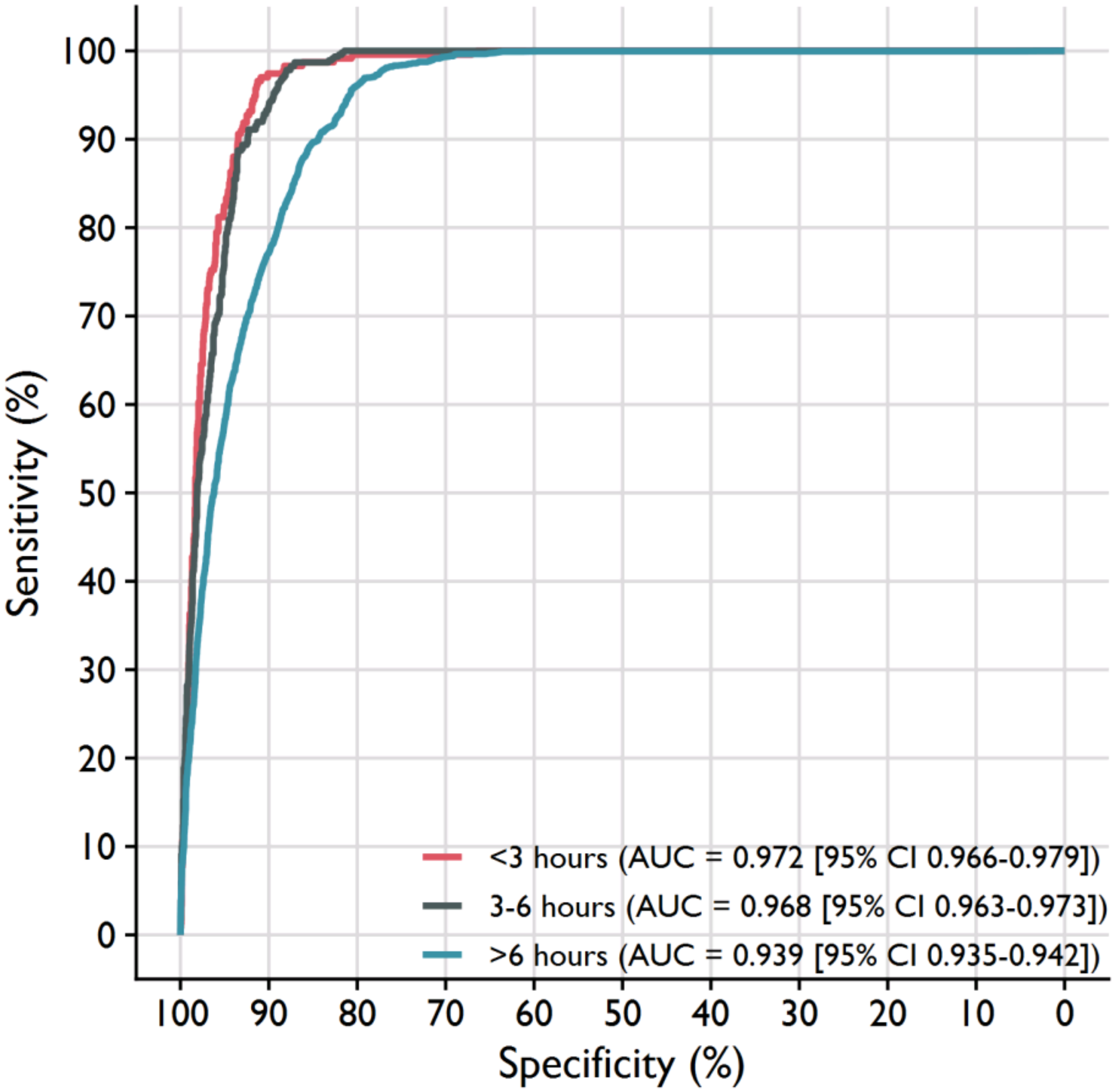

Supplemental Figure S7.

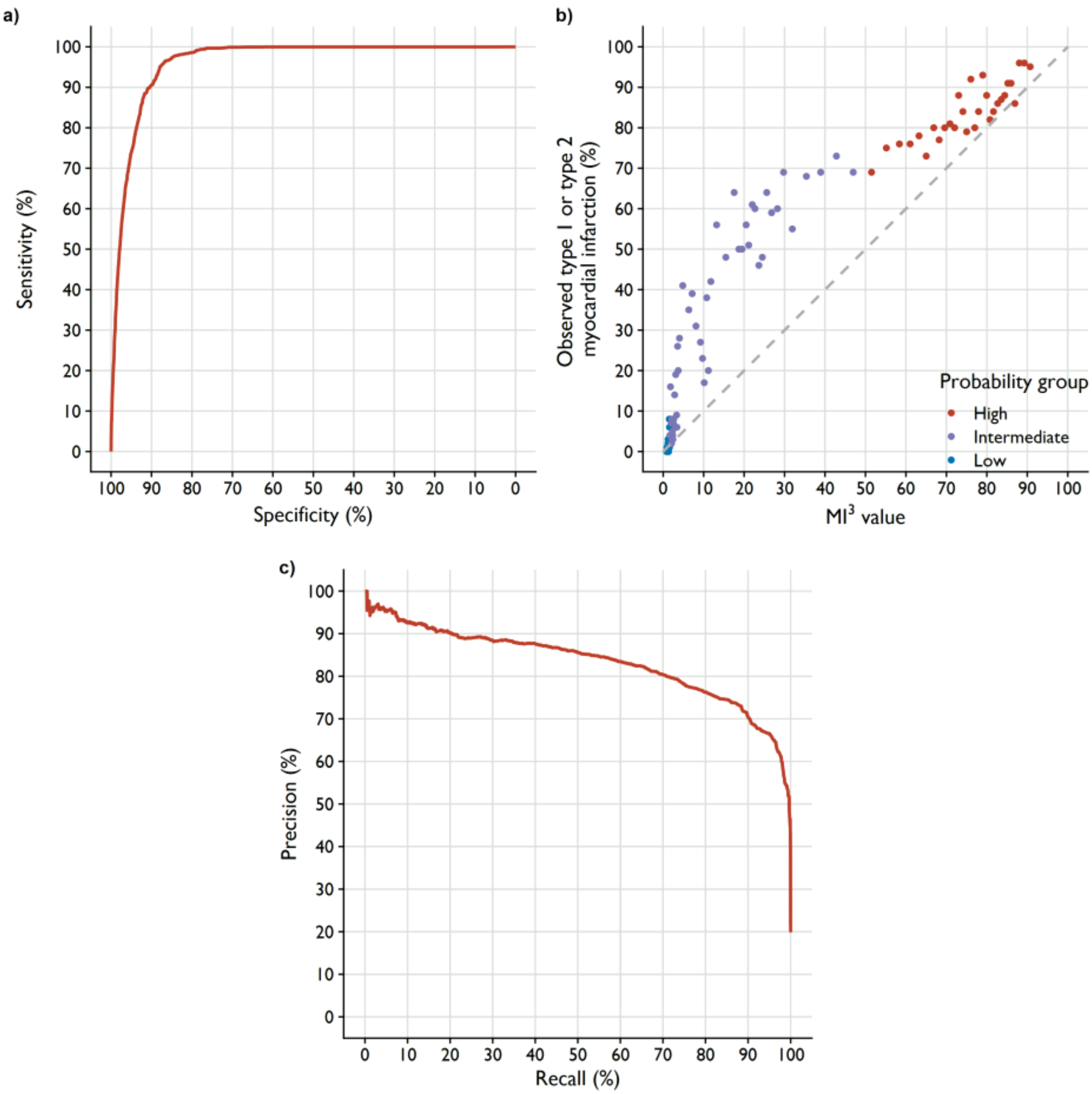

Supplemental Figure S8.

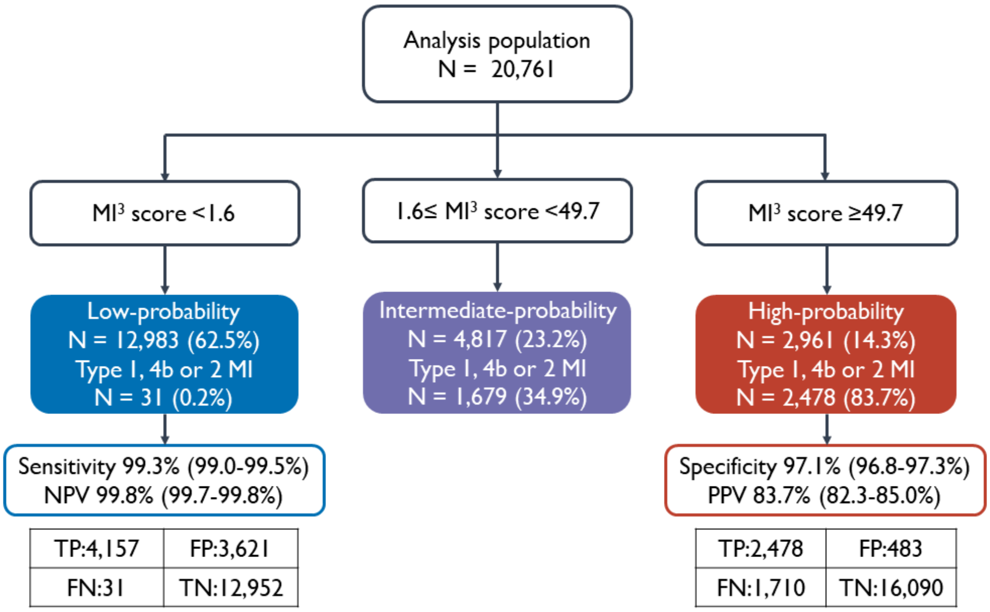

Supplemental Table S1.

	Example MI^3 threshold	TN	FN	TP	FP	Sensitivity (%)	NPV (%)	Specificity (%)	PPV (%)
**Low probability**	1.6	12,961	22	3,250	4,528	99.3 (99.0-99.6)	99.8 (99.8-99.9)	74.1 (73.5-74.8)	41.8 (41.2-42.4)
**High Probability**	49.7	16,612	1,188	2,084	877	63.7 (62.2-65.3)	93.3 (93.1-93.6)	95.0 (94.6-95.3)	70.4% (68.7-72.0)

Supplemental Table S2.

	**Testing set**					**Validation set**				
**Example MI^3 threshold**	**Sensitivity (%)**	**NPV (%)**	**Specificity (%)**	**PPV (%)**	**Proportion ruled out / ruled in (%)**	**Sensitivity (%)**	**NPV (%)**	**Specificity (%)**	**PPV (%)**	**Proportion ruled out / ruled in (%)**
**1.6**	97.8 (96.8-98.7)	99.7 (99.5-99.8)	77.4 (76.4-78.4)	33.9 (32.0-35.8)	69%	99.3 (99.0-99.6)	99.8 (99.8-99.9)	74.1 (73.5-74.8)	41.8 (41.2-42.4)	63%
**49.7**	71.5 (68.4-74.3)	96.6 (96.2-97.0)	96.7 (96.3-97.1)	71.7 (68.9-74.8)	11%	63.7 (62.2-65.3)	93.3 (93.1-93.6)	95.0 (94.6-95.3)	70.4 (68.7-72.0)	14%

Validation of a machine learning algorithm to guide the diagnosis of myocardial infarction

Study Investigators:
Professor Nicholas Mills, University of Edinburgh
Dr Martin Than, Christchurch Hospital
Professor John Pickering, University of Otago
Dr Ken Lee, University of Edinburgh
Mr Dimitrios Doudesis, University of Edinburgh

Statistical analysis plan. Version 2 (May 10th 2020)

Contents

Contents **13**

1 Introduction **14**

1.1 Background *14*

1.2 Study questions *14*

2 Objectives **15**

3 Statistical analysis plan: External validation of the MI^3 machine learning algorithm **15**

3.1 Study design *15*

3.2 Primary endpoint *15*

3.3 Secondary endpoints *15*

3.4 List of analyses *15*

3.4.1 Baseline characteristics 15

3.4.2 Event summary 16

3.4.3 Evaluating the MI^3 machine learning algorithm for the diagnosis of acute myocardial infarction 17

3.4.4 Secondary analysis 17

4 References **18**

1 Introduction

1.1 Background

Chest pain is one of the most common reasons for presentation to hospital worldwide, with more than one million attendances each year in the United Kingdom alone[1]. Despite improvements in diagnosis and treatment of myocardial infarction, and major reductions in 30-day mortality over the last 10 years, approximately 70,000 patients die each year. Prompt recognition of patients with acute coronary syndrome is crucial to guide correct investigation and management with direct impact on clinical outcomes.

Accelerated diagnostic pathways aim to promote earlier discharge in those considered low-risk and improve the targeting of treatment to high-risk patients.[2-5] However, these pathways have some limitations. First, they use fixed cardiac troponin thresholds for all patients, which do not account for age or comorbidities that are known to influence troponin concentrations.[5, 6] Second, they are based on fixed time-points for serial testing, which can be challenging in a busy Emergency Department, and such pathways may not be generalisable to all health care systems. Third, they broadly categorise patients as either low-, intermediate- or high-risk, which does not necessarily reflect the continuum of risk.

The myocardial-ischemic-injury-index (MI^3) is a machine learning algorithm developed using gradient boosting, to compute an individualised probability of myocardial infarction on a scale of 0-100 for patients with suspected acute coronary syndrome.[7] The MI^3 score is calculated using age, sex and two cardiac troponin concentrations.

Using the large High-STEACS[8] database of consecutive patients with suspected acute coronary syndrome, we will externally validate this machine learning algorithm in a more heterogeneous patient population.

1.2 Study questions

1. What is the diagnostic performance and clinical utility of the MI^3 machine learning algorithm to *exclude* or *confirm* a diagnosis of acute myocardial infarction?

2. What is the cardiovascular risk beyond the initial diagnosis of patients stratified by the MI^3 machine learning algorithm at one year?

2 Objectives

The objective of this SAP is to describe the statistical analysis contributing to the final report of the analysis described below.

3 Statistical analysis plan: External validation of the MI^3 machine learning algorithm

3.1 Study design

Using data from the High-Sensitivity Troponin in the Evaluation of patients with suspected Acute Coronary Syndrome (High-STEACS) trial,[8] we will evaluate the diagnostic performance of the MI^3 machine learning algorithm for the diagnosis of acute myocardial infarction.

3.2 Primary endpoint

- The primary endpoint will be acute myocardial infarction (type 1 or type 4b) at index hospitalisation.

3.3 Secondary endpoints

- Acute myocardial infarction (type 1, type 2 or type 4b) at index hospitalisation
- Acute myocardial infarction (type 1 or type 4b) or cardiovascular death at 1 year
- All-cause death at 1 year

3.4 List of analyses

3.4.1 Baseline characteristics

Summary statistics will be provided for the baseline characteristics for the study population. The following variables will be reported:

- Age (years), median (interquartile range, IQR)
- Sex (women/men), n (%)
- Presenting symptom, n (%)
  - Chest pain
  - Dyspnoea
  - Palpitation
  - Syncope
  - Other

- Past medical history , n (%)
  - Ischaemic heart disease
  - Myocardial infarction
  - Diabetes mellitus
  - Cerebrovascular disease
- Previous revascularisation, n (%)
  - Percutaneous coronary intervention
  - Coronary artery bypass grafting
- Medications at presentation, n (%)
  - Aspirin
  - Dual anti-platelet therapy
  - Statin
  - Angiotensin converting enzyme inhibitor or angiotensin receptor blockers
  - Beta-blocker
  - Oral anticoagulant
- Haematology and clinical chemistry measurements, median (interquartile range, IQR)
  - Haemoglobin, g/L
  - Estimated glomerular filtration rate, mL/min
  - Peak high-sensitivity cardiac troponin, ng/L

3.4.2 Event summary

The analyses will be presented stratified for the following event groups

- Adjudicated diagnosis of acute myocardial infarction (type 1 or type 4b) at index hospitalisation
- Adjudicated diagnosis of acute myocardial infarction (type 1, type 2 or type 4b) at index hospitalisation
- Adjudicated diagnosis of acute myocardial infarction (type 1 or type 4b) or cardiovascular death at 1 year
- All-cause death at 1 year

3.4.3 Evaluating the MI^3 machine learning algorithm for the diagnosis of acute myocardial infarction

We will evaluate the sensitivity, specificity, negative predictive value (NPV), positive predictive value (PPV) and the proportion of patients identified by the MI^3 machine learning algorithm to *rule-out* and *rule-in* the primary outcome.[9] We will subsequently evaluate the diagnostic performance across a range of pre-specified subgroups as follows:

- Age
- Sex
- Renal function
- Past medical history (presence or absence of ischaemic heart disease, myocardial infarction, diabetes, cerebrovascular disease)
- Time from onset of symptoms to presentation

3.4.4 Secondary analysis

- Diagnostic performance for composite outcomes including type 1, type 2 or type 4b at index hospitalisation
- Evaluate performance based on the time interval between blood sampling.
- Diagnostic performance for composite outcomes including type 1, type 2 or type 4b or cardiovascular death at 1 year and for all-cause death at 1 year

4 References

1. Sanchis-Gomar F, Perez-Quilis C, Leischik R, Lucia A. Epidemiology of coronary heart disease and acute coronary syndrome. Ann Transl Med 2016; 4(13): 256.

2. Body R, Carlton E, Sperrin M, Lewis PS, Burrows G, Carley S, McDowell G, Buchan I, Greaves K and Mackway-Jones K. Troponin-only Manchester Acute Coronary Syndromes (T-MACS) decision aid: single biomarker re-derivation and external validation in three cohorts. *Emerg Med J*. 2017;34:349-356.

3. Reichlin T, Schindler C, Drexler B, Twerenbold R, Reiter M, Zellweger C, Moehring B, Ziller R, Hoeller R, Rubini Gimenez M, Haaf P, Potocki M, Wildi K, Balmelli C, Freese M, Stelzig C, Freidank H, Osswald S and Mueller C. One-hour rule-out and rule-in of acute myocardial infarction using high-sensitivity cardiac troponin T. *Arch Intern Med*. 2012;172:1211-8.

4. Than M, Aldous S, Lord SJ, Goodacre S, Frampton CMA, Troughton R, George P, Florkowski CM, Ardagh M, Smyth D, Jardine DL, Peacock WF, Young J, Hamilton G, Deely JM, Cullen L and Richards AM. A 2-Hour Diagnostic Protocol for Possible Cardiac Chest Pain in the Emergency Department. *JAMA Internal Medicine*. 2014;174:51.

5. Chapman AR, Anand A, Boeddinghaus J, Ferry AV, Sandeman D, Adamson PD, Andrews J, Tan S, Cheng SF, D'Souza M, Orme K, Strachan FE, Nestelberger T, Twerenbold R, Badertscher P, Reichlin T, Gray A, Shah ASV, Mueller C, Newby DE and Mills NL. Comparison of the Efficacy and Safety of Early Rule-Out Pathways for Acute Myocardial Infarction. *Circulation*. 2017;135:1586-1596.

6. Thygesen K, Alpert JS, Jaffe AS, Simoons ML, Chaitman BR, White HD, Joint ESCAAHAWHFTFftUDoMI, Katus HA, Lindahl B, Morrow DA, Clemmensen PM, Johanson P, Hod H, Underwood R, Bax JJ, Bonow RO, Pinto F, Gibbons RJ, Fox KA, Atar D, Newby LK, Galvani M, Hamm CW, Uretsky BF, Steg PG, Wijns W, Bassand JP, Menasche P, Ravkilde J, Ohman EM, Antman EM, Wallentin LC, Armstrong PW, Simoons ML, Januzzi JL, Nieminen MS, Gheorghiade M, Filippatos G, Luepker RV, Fortmann SP, Rosamond WD, Levy D, Wood D, Smith SC, Hu D, Lopez-Sendon JL, Robertson RM, Weaver D, Tendera M, Bove AA, Parkhomenko AN, Vasilieva EJ and Mendis S. Third universal definition of myocardial infarction. *Circulation*. 2012;126:2020-35.

7. Than MP, Pickering JW, Sandoval Y, Shah ASV, Tsanas A, Apple FS, Blankenberg S, Cullen L, Mueller C, Neumann JT, Twerenbold R, Westermann D, Beshiri A, Mills NL and collaborative MI. Machine Learning to Predict the Likelihood of Acute Myocardial Infarction. *Circulation*. 2019;140:899-909.

8. Shah ASV, Anand A, Strachan FE, Ferry AV, Lee KK, Chapman AR, Sandeman D, Stables CL, Adamson PD, Andrews JPM, Anwar MS, Hung J, Moss AJ, O'Brien R, Berry C, Findlay I, Walker S, Cruickshank A, Reid A, Gray A, Collinson PO, Apple FS, McAllister DA, Maguire D, Fox KAA, Newby DE, Tuck C, Harkess R, Parker RA, Keerie C, Weir CJ, Mills NL and High SI. High-sensitivity troponin in the evaluation of patients with suspected acute coronary syndrome: a stepped-wedge, cluster-randomised controlled trial. *Lancet*. 2018;392:919-928.

9. Collet J-P, Thiele H, Barbato E, Barthélémy O, Bauersachs J, Bhatt DL, Dendale P, Dorobantu M, Edvardsen T, Folliguet T, Gale CP, Gilard M, Jobs A, Jüni P, Lambrinou E,

Lewis BS, Mehilli J, Meliga E, Merkely B, Mueller C, Roffi M, Rutten FH, Sibbing D, Siontis GCM, Kastrati A, Mamas MA, Aboyans V, Angiolillo DJ, Bueno H, Bugiardini R, Byrne RA, Castelletti S, Chieffo A, Cornelissen V, Crea F, Delgado V, Drexel H, Gierlotka M, Halvorsen S, Haugaa KH, Jankowska EA, Katus HA, Kinnaird T, Kluin J, Kunadian V, Landmesser U, Leclercq C, Lettino M, Meinila L, Mylotte D, Ndrepepa G, Omerovic E, Pedretti RFE, Petersen SE, Petronio AS, Pontone G, Popescu BA, Potpara T, Ray KK, Luciano F, Richter DJ, Shlyakhto E, Simpson IA, Sousa-Uva M, Storey RF, Touyz RM, Valgimigli M, Vranckx P, Yeh RW, Barbato E, Barthélémy O, Bauersachs J, Bhatt DL, Dendale P, Dorobantu M, Edvardsen T, Folliguet T, Gale CP, Gilard M, Jobs A, Jüni P, Lambrinou E, Lewis BS, Mehilli J, Meliga E, Merkely B, Mueller C, Roffi M, Rutten FH, Sibbing D, Siontis GCM, Kastrati A, Mamas MA, Aboyans V, Angiolillo DJ, Bueno H, Bugiardini R, Byrne RA, Castelletti S, Chieffo A, Cornelissen V, Crea F, Delgado V, Drexel H, Gierlotka M, Halvorsen S, Haugaa KH, Jankowska EA, Katus HA, Kinnaird T, Kluin J, Kunadian V, Landmesser U, Leclercq C, Lettino M, Meinila L, Mylotte D, Ndrepepa G, Omerovic E, Pedretti RFE, Petersen SE, Petronio AS, Pontone G, Popescu BA, Potpara T, Ray KK, Luciano F, Richter DJ, Shlyakhto E, Simpson IA, Sousa-Uva M, Storey RF, Touyz RM, Valgimigli M, Vranckx P, Yeh RW, Barbato E, Barthélémy O, Bauersachs J, Bhatt DL, Dendale P, Dorobantu M, Edvardsen T, Folliguet T, Gale CP, Gilard M, Jobs A, Jüni P, Lambrinou E, Lewis BS, Mehilli J, Meliga E, Merkely B, Mueller C, Roffi M, Rutten FH, Sibbing D and Siontis GCM. 2020 ESC Guidelines for the management of acute coronary syndromes in patients presenting without persistent ST-segment elevation. *European Heart Journal*. 2020.
